# The importance of social networks in neurosurgery training in low/middle income countries

**DOI:** 10.3389/fsurg.2024.1341148

**Published:** 2024-03-13

**Authors:** Manuel de Jesus Encarnacion Ramirez, Jeff Natalaja Mukengeshay, Gennady Chumtin, Renat Nurmukhametov, Matias Baldoncini, Jesus Lafuente, Andreina Rosario Rosario, Siddarth Kannan, Aderehime Haidara, Issael Ramirez, Ismail Bozkurt, Ignatius Esene, Stanislav Kaprovoy, Nikolay Konovalov, Kazadi Kelvin Kalangu, Gerald Musa, Michael T. Lawton, Vishal K. Chavda, Eric Suero Molina, Nicola Montemurro

**Affiliations:** ^1^ Department of Neurosurgery, Russian People’s Friendship University, Moscow, Russia; ^2^ Department Neurosurgery, Clinique Ngaliema, Kinshasa, Democratic Republic of Congo; ^3^ Laboratory of Microsurgical Neuroanatomy, Second Chair of Gross Anatomy, School of Medicine, University of Buenos Aires, Buenos Aries, Argentina; ^4^ Spine Center Hospital del Mar, Sagrat Cor University Hospital, Barcelona, Spain; ^5^ Medical Student, Autonomous University of Santo Domingo (UASD), Santo Domingo, Dominican Republic; ^6^ School of Medicine, University of Central Lancashire, Preston, United Kingdom; ^7^ Department of Neurosurgery, Service de neurochirurgie CHU de Bouaké, Bouake, Côte d'Ivoire; ^8^ Department of Neurosurgery, Royal Melbourne Hospital, Melbourne, VIC, Australia; ^9^ Department of Neurosurgery, Medical Park Ankara Hospital, Ankara, Turkiye; ^10^ Department of Neurosurgery, Yuksek Ihtisas University, School of Medicine, Ankara, Turkiye; ^11^ Neurosurgery Division, Faculty of Health Sciences, University of Bamenda, Bambili, Cameroon; ^12^ Department of Neurosurgery, N.N. Burdenko National Medical Research Center of Neurosurgery, Moscow, Russia; ^13^ Department of Neurosurgery, College of Health Sciences, University of Zimbabwe, Harare, Zimbabwe; ^14^ Neurosurgery Resident, Department of Neurosurgery, People's Friendship University of Russia, Moscow, Russia; ^15^ Department of Neurosurgery, Barrow Neurological Institute, St. Joseph's Hospital and Medical Centre, Phoenix, AZ, United States; ^16^ Department of Medicine, Multispeciality, Trauma and ICCU Center, Sardar Hospital, Gujarat, India; ^17^ Department of Neurosurgery, University Hospital of Muenster, Münster, Germany; ^18^ Department of Neurosurgery, Azienda Ospedaliero Universitaria Pisana (AOUP), University of Pisa, Pisa, Italy

**Keywords:** social media, social networks, neurosurgery, survey, training

## Abstract

**Introduction:**

Neurosurgery is evolving with new techniques and technologies, relies heavily on high-quality education and training. Social networks like Twitter, Facebook, Instagram and LinkedIn have become integral to this training. These platforms enable sharing of surgical experiences, fostering global knowledge-sharing and collaboration among neurosurgeons. Virtual conferences and courses are accessible, enhancing learning regardless of location. While these networks offer real-time communication and collaborative opportunities, they also pose challenges like the spread of misinformation and potential distractions. According to the PICO format, the target population (P) for the purpose of this paper are medical students, neurosurgical residents and consultants on the role of social media (I) in neurosurgery among Low-Middle income countries (C) with the main outcome to understand the collaborative domain of learning.

**Material and method:**

This cross-sectional survey, conducted in June-July 2023, involved 210 medical students, neurosurgery residents, fellows, and practicing neurosurgeons from low and middle-income countries. A structured questionnaire assessed social network usage for neurosurgery training, covering demographic details, usage frequency, and purposes like education, collaboration, and communication. Participants rated these platforms' effectiveness in training on a 1–5 scale. Data collection employed emails, social media groups, and direct messaging, assuring respondent anonymity. The survey aimed to understand and improve social networks' use in neurosurgery, focusing on professional development, challenges, and future potential in training.

**Results:**

In a survey of 210 participants from low and middle-income countries, 85.5% were male, 14.5% female, with diverse roles: 42.9% neurosurgery residents, 40% practicing neurosurgeons, 14.6% medical students, and 2.4% other healthcare professionals. Experience ranged from 0 to 35 years, with Mexico, Nigeria, and Kenya being the top participating countries. Most respondents rated neurosurgery training resources in their countries as poor or very poor. 88.7% used social media professionally, predominantly WhatsApp and YouTube. Content focused on surgical videos, research papers, and webinars. Concerns included information quality and data privacy. Interactive case discussions, webinars, and lectures were preferred resources, and most see a future role for social media in neurosurgery training.

**Conclusions:**

Our study underscores the crucial role of social media in neurosurgery training and practice in low and middle-income countries (LMICs). Key resources include surgical videos, research papers, and webinars. While social media offers a cost-effective, global knowledge-sharing platform, challenges like limited internet access, digital literacy, and misinformation risks remain significant in these regions.

## Introduction

The field of neurosurgery is an art constantly changing, with the advent of new techniques, tools, and technologies being developed to improve patient outcomes. For neurosurgeons to stay updated with these advancements and continuously improve their skills, they must have access to high-quality education and training opportunities. One essential tool that has emerged as a valuable resource for neurosurgery training are the social networks.

Social networks, such as Twitter and Facebook, have become increasingly popular and revolutionized how we communicate and share information. In the medicine field, social networks have been used to facilitate communication and collaboration between healthcare professionals, as well provide access to medical knowledge and training resources. In addition to Twitter and Facebook, other social networks have emerged as valuable tools for neurosurgery training. One such platform is Instagram, which has gained popularity among medical professionals due to its visual nature (1, 2). Neurosurgeons can use Instagram to share images and videos of surgical procedures, neuroanatomy, and pathology, allowing for a more engaging and interactive learning experience. Instagram also offers the opportunity to connect with other neurosurgeons and medical professionals worldwide, creating a global network of knowledge-sharing and collaboration (3). LinkedIn is primarily known as a professional networking site, it also offers a range of educational resources, including online courses, webinars, and discussion forums. Neurosurgery trainees can use LinkedIn to connect with mentors and experts in the field (4).

YouTube is another social network that has become increasingly important in neurosurgery training. The platform offers a vast library of educational audiovisual content on a range of topics, from anatomy and physiology to surgical techniques and procedures (5). Neurosurgery trainees can use YouTube to access tutorials, lectures, and case presentations, allowing for a more immersive and self-directed learning experience. The platform also offers the opportunity to connect with other medical professionals and to collaborate on research projects and case studies (6).

WhatsApp and Telegram have been used to create groups for neurosurgeons to share interesting cases, discuss complex surgical procedures, and seek advice from peers, e.g., Neurosurgerycoach and neurosurgery cocktail. These groups have facilitated collaboration and have allowed for a wider dissemination of knowledge and experience (7, 8). Furthermore, online platforms such as Neurosurgery TV have emerged as a valuable resource for neurosurgery training. Neurosurgery TV provides access to educational content, surgical videos, and webinars, allowing neurosurgeons to learn from experts and stay updated with the latest advances in neurosurgery.

Through social networks, neurosurgeons can collaborate on research projects. Additionally, social networks can provide access to virtual conferences and educational courses, allowing neurosurgeons to learn from experts in their field regardless of their geographic location (9). One of the principal advantages of social networks in neurosurgery training is their possibility of facilitating real-time communication and collaboration. For example, during complex surgical procedures, neurosurgeons can use social networks to share information and receive feedback from their peers, helping to improve patient outcomes and decrease the risk of complications. Additionally, social networks can supply a platform for neurosurgeons to discuss difficult cases, request advice from colleagues, and receive support from their peers (10, 11).

Despite the countless benefits of social networks in neurosurgery training, there are also some challenges that need to be addressed. For example, there is a risk that social networks could be used to spread disinformation or recommend unproven treatment methods. Additionally, social networks can be a source of distraction and could potentially detract from the quality of patient care if used improperly (12, 13).

In order to address these challenges, it is crucial for neurosurgeons to use social networks with responsibility and verify the accuracy of all information they encounter online. Additionally, neurosurgeons should be aware of the potential risks associated with social networks and take steps to minimize these risks, such as by limiting the amount of time spent on social networks during clinical hours (10).

Social networks like Twitter, Facebook, WhatsApp, Telegram, VK, and NeurosurgeryTV play a crucial role in neurosurgery training by providing a platform for communication, collaboration, and access to information and training resources (Table 1) (14). The benefits of social networks in neurosurgery training cannot be overstated, and it is essential for neurosurgeons to embrace these technologies to boost their skills, enhance patient care, and advance the field of neurosurgery as a whole. There are some challenges associated with social networks, when used responsibly, they can be an asset advantage for neurosurgeons looking to improve their clinical skills and knowledge (8, 15). As the field of neurosurgery continues to evolve, social networks are likely to become an increasingly predominant tool for education and professional development.

**Table 1:** Advantages & disadvantages of social media platforms.

Social media platform	Advantages	Disadvantages
WhatsApp	1. Ease of use 2. Cross-platform compatibility 3. Group messaging 4. Video and voice calls	1. Media storage limitations 2. Security vulnerabilities 3. Limited networking opportunities
Facebook	1. Ease of use 2. Easy to share information 3. Networking opportunities 4. Community building	1. Relatively less used compared to other platforms 2. No video or voice calls
Twitter	1. Real-Time Information 2. Global reach 3. Networking opportunities 4. Platform to share research	1. No video or voice calls
Instagram	1. Patient education via videos 2. Community engagement 3. Video and voice calls	1. Privacy concerns 2. Limited networking opportunities
YouTube	1. Patient education via videos 2. Global reach	1. Limited networking opportunities 2. Misinformation
LinkedIn	1. Networking 2. Career development 3. Reputation management 4. Professional branding	1. Network saturation 2. Job search challenges
VK	1. Ease of use 2. Easy to share information 3. Networking opportunities 4. Community building	1. Relatively less used compared to other platforms 2. No video or voice calls 3. Only used in Russia

According to the PICO format, the target population (P) for the purpose of this paper are medical students, neurosurgical residents and consultants on the role of social media (I) in neurosurgery among Low-Middle income countries (C) with the main outcome to understand the collaborative domain of learning.

## Materials and methods

### Design and sample

This study was a cross-sectional survey conducted between June and July 2023. The sample population consisted of medical students with interest in neurosurgery, neurosurgery residents, fellows, and practicing neurosurgeons from low and middle-income countries (LMICs). The total number of participants reached was 210.

### Instrument

A structured questionnaire was designed to evaluate the use of social networks for neurosurgery training (Table 2). The questionnaire consisted of demographic information such as age, gender, years of practice, and country of practice. It also included questions regarding the usage frequency of different social networks, purpose of use (educational/collaboration/communication), the effectiveness of these platforms in neurosurgery training, perceived benefits and challenges, and suggestions for improvement.

**Table 2:** Survey on the use of social networks in neurosurgery.

Survey on the Use of Social Networks in Neurosurgery
Section 1: Personal Information
Gender: Male, Female, Other, Prefer not to say)
Age
Country of Residence
Current Occupation: Neurosurgeon, Neurosurgery Resident, Medical Student, Other—Please specify)
Years of Experience in Neurosurgery
Do you work in a rural or urban setting? (Rural, Urban)
Section 2: Access to Neurosurgery Training Resources
How would you rate the availability of neurosurgery training resources in your country? (Very Poor, Poor, Average, Good, Excellent)
What challenges do you face in accessing neurosurgery training resources in your country? (Open-ended question)
Section 3: Usage of Social Media for Professional Purposes
Do you use social media for professional purposes in the field of neurosurgery? (Yes, No)
If yes, which social media platforms do you use most frequently for this purpose? (Facebook, Twitter, LinkedIn, Instagram, Other—Please specify)
What types of content do you typically seek or share on social media related to neurosurgery? (Research papers, Clinical case discussions, Surgical technique videos, Webinars, Other—Please specify)
Section 4: Role of Social Media in Neurosurgery Training
On a scale of 1 (not at all) to 5 (very much), how much has social media contributed to your professional development in neurosurgery?
Can you provide any specific instances where social media has significantly contributed to your neurosurgery learning or practice? (Open-ended question)
Have you ever participated in a neurosurgery webinar or online course facilitated through a social media platform? (Yes, No)
Have you ever sought or received advice on a challenging case from colleagues on social media? (Yes, No)
Section 5: Challenges and Concerns
What are the challenges you have faced while using social media for neurosurgery training? (Open-ended question)
Do you have any concerns about the use of social media in neurosurgery training (e.g., data privacy, quality of information)? (Open-ended question)
Section 6: Future Potential
How do you see the role of social media in neurosurgery training evolving in the future? (Open-ended question)
What steps could be taken to maximize the benefits of social media for neurosurgery training in low-income countries? (Open-ended question)
Section 7: Perceived Benefits and Challenges
What do you see as the main benefits of using social networks for professional purposes in neurosurgery? (Access to global expertise, Improved knowledge sharing, Greater networking opportunities, Enhanced access to training resources, Other—Please specify)
What do you see as the main challenges of using social networks for professional purposes in neurosurgery? (Privacy concerns, Quality of information, Time management, Professional boundaries, Other—Please specify)
Have you ever encountered any negative consequences or ethical issues related to your use of social networks in neurosurgery practice? If yes, please describe. (Open-ended question)
Section 8: Training and Education
Have you ever used social networks for neurosurgery training or continuing education? If yes, please describe your experience. (Open-ended question)
Would you be interested in using social networks for neurosurgery training or continuing education in the future? (Yes, No)
If yes, what types of resources would you find most useful? (Select all that apply: Live webinars, Recorded lectures, Interactive case discussions, Journal clubs, Other—Please specify)

The questions related to social networks use included a rating scale to evaluate how frequently participants used these platforms (daily, weekly, monthly, rarely, or never) and for what purposes (learning new techniques, discussing cases, staying updated with the latest research, etc.). Participants were also asked to rate the effectiveness of social networks on their neurosurgery training on a scale of 1–5, with 1 being not effective and 5 being extremely effective.

### Data collection

The online survey was distributed via various channels. These included email lists of neurosurgery societies in LMICs, social media groups, and direct messaging to professionals known to the researchers. Participation was voluntary, and respondents were assured of their anonymity and the confidentiality of their responses.

## Results

### Demographics

**Figure 1:**
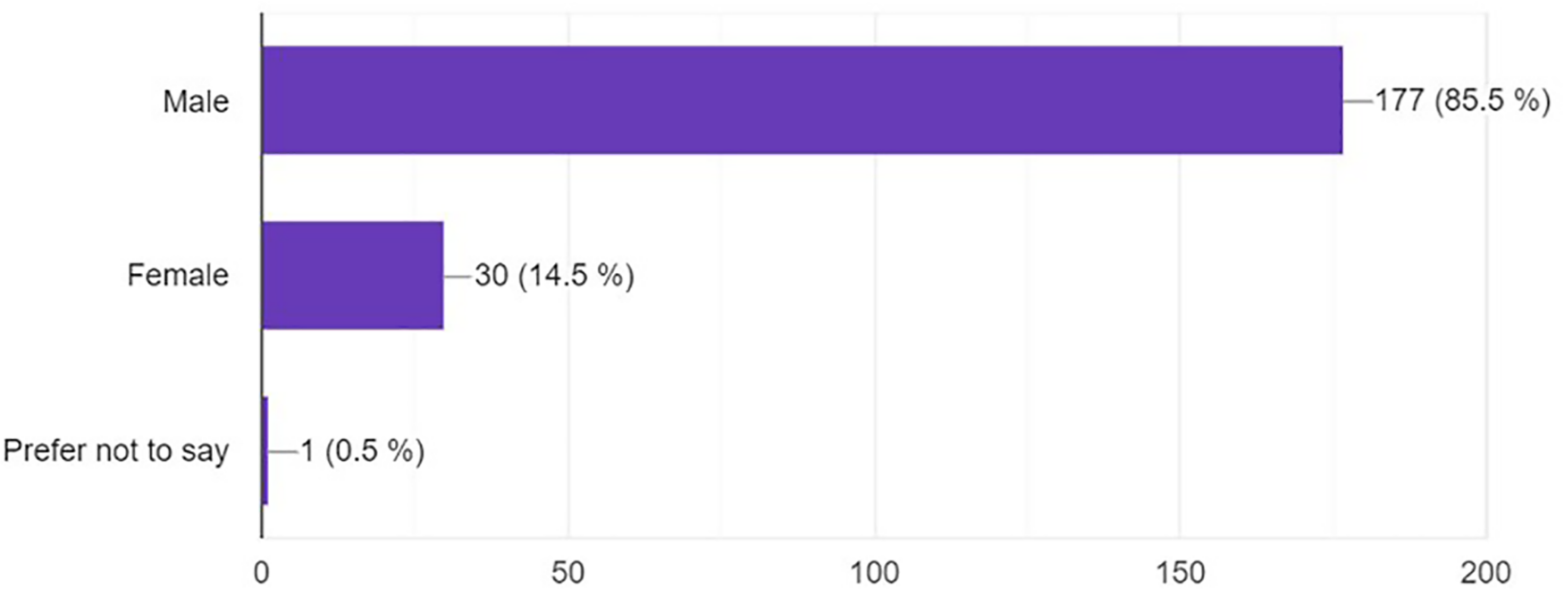
Demographic of patients answered the questionnaire.

The vast majority of respondents (85.5%) were male, while 14.5% were female, and one respondent (0.5%) preferred not to disclose their gender (Figure 1).

**Figure 2:**
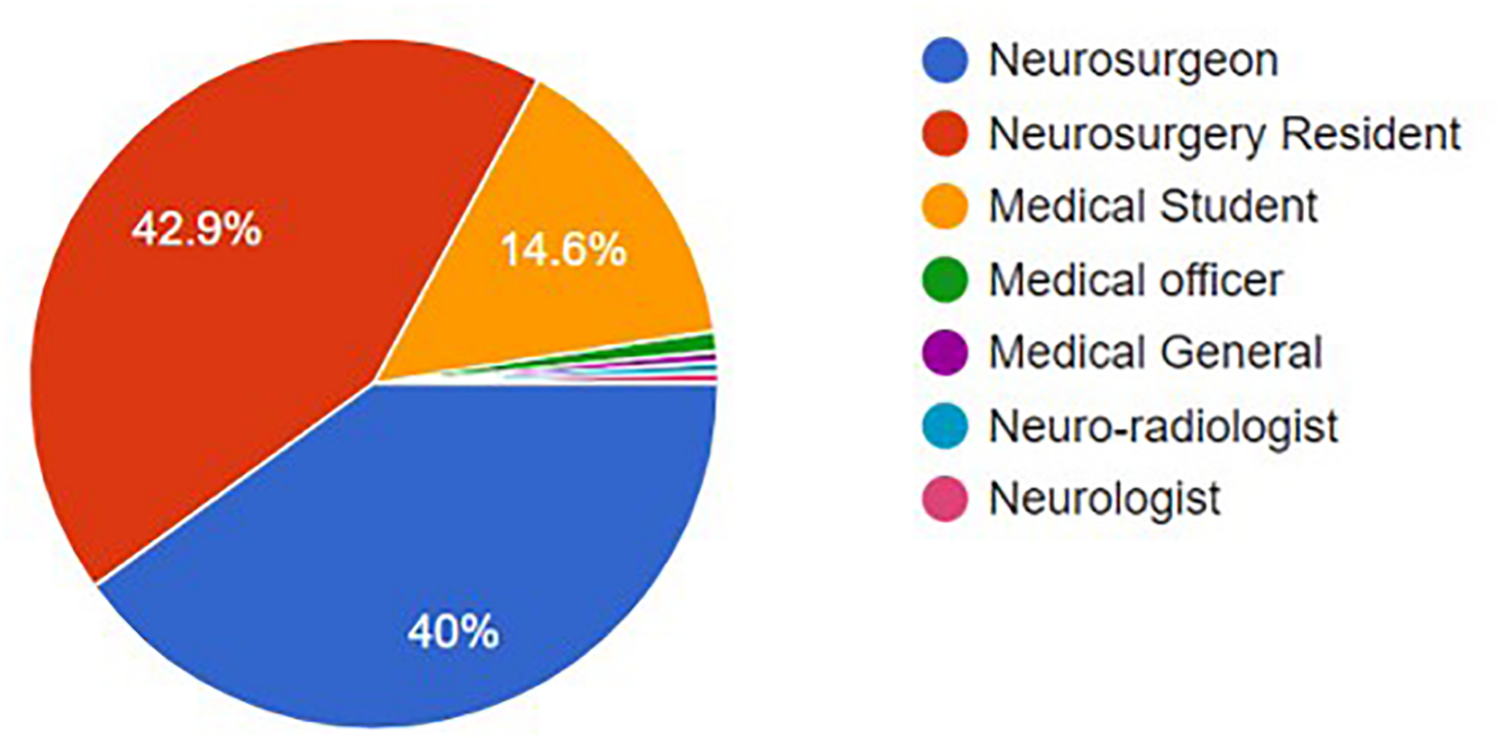
Type of professionals answered the survey.

The highest proportion of respondents were neurosurgery residents (42.9%), closely followed by practicing neurosurgeons (40%). Medical students accounted for 14.6% of the sample, and the remaining 2.4% were other healthcare professionals including a medical officer, medical general, neuro-radiologist, and neurologist (Figure 2).

**Figure 3:**
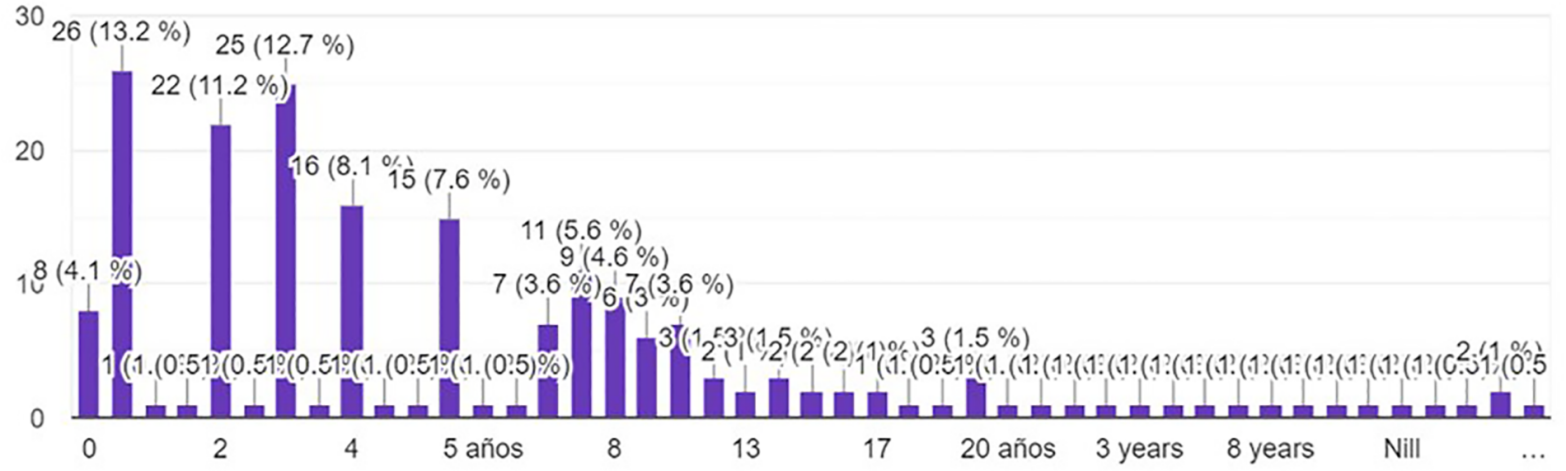
Experience of responders in neurosurgery.

The largest group of respondents had 1 year of experience in neurosurgery (13.2%), followed by those with 3 years of experience (12.7%). The remainder of the respondents had varied years of experience ranging from 0 to 35 years, with 13.3% of them not answering this question (Figure 3).

Mexico takes the lead with 24, followed by Nigeria with 19 and Kenya at 17. Brazil, Dominican Republic, and the Russian Federation each have a substantial representation of 14. Countries like India and Ethiopia exhibit moderate numbers, with 9 each. A significant portion of countries, including Belarus, Botswana, Congo DR, Haiti, Madagascar, Malaysia, Mozambique, Nicaragua, Panama, Salvador, South Africa, Syria, Tajikistan, and Uganda have the least representation with only one entity each. This distribution suggests varying levels of engagement or presence across diverse global regions, with certain countries showcasing heightened activity or participation and others, minimal to moderate involvement.

African countries seem to be well-represented with multiple nations from the continent appearing in the list, ranging from high representation (like Kenya and Nigeria) to single counts (like Uganda or Mozambique).

Similarly, Latin American countries have varying degrees of representation, from Mexico's highest count to singular representations like Panama.

## Access to training resources and use of social Media

**Figure 4:**
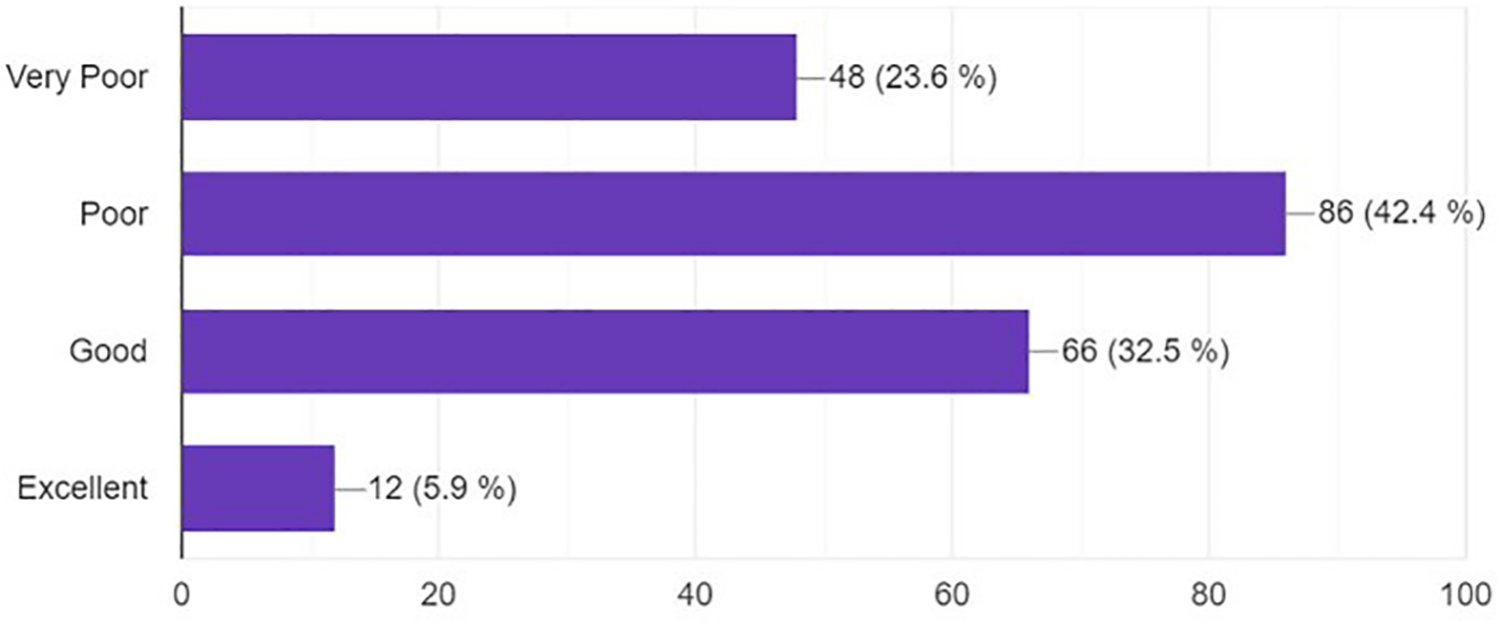
Bar graph demonstrating type of neurosurgery training resources on social media.

Most of the respondents felt the availability of neurosurgery training resources in their countries was poor (42.4%) or very poor (23.6%), while 32.5% rated it as good, and a minority (5.9%) as excellent (Figure 4).

**Figure 5:**
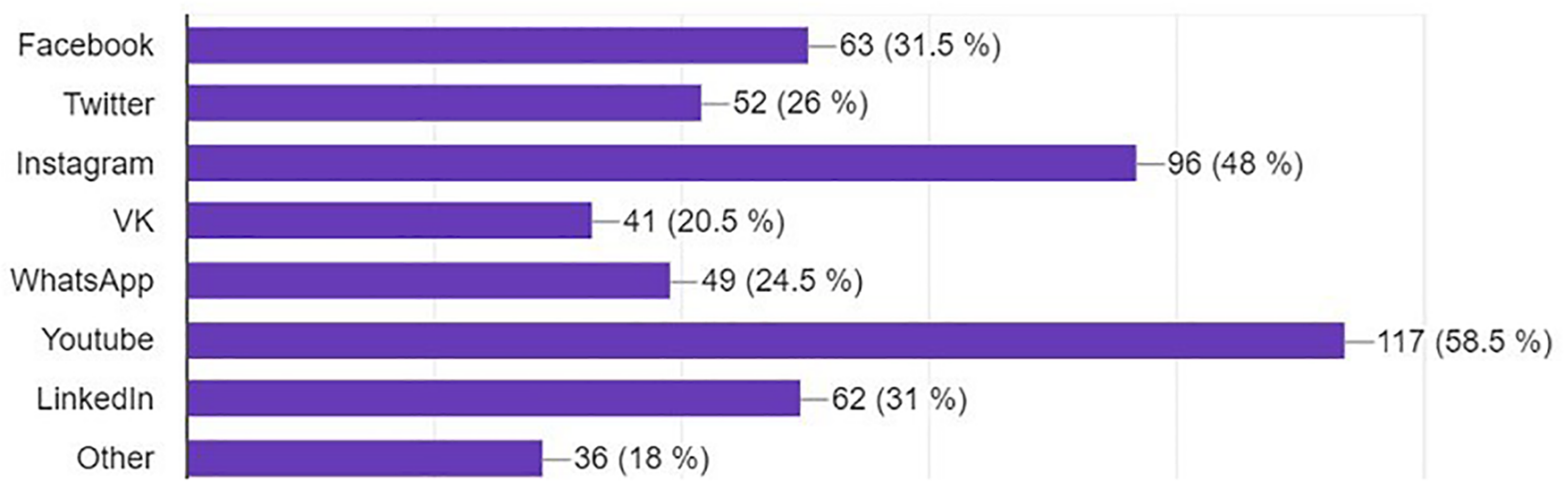
Bar graph demonstrating most used platforms for professional use.

The vast majority (88.7%) reported using social media for professional purposes in the field of neurosurgery. The most popular platforms were WhatsApp and YouTube (both at 58%), followed by Instagram (48%) (Figure 5).

### Content sought on social media

**Figure 6:**
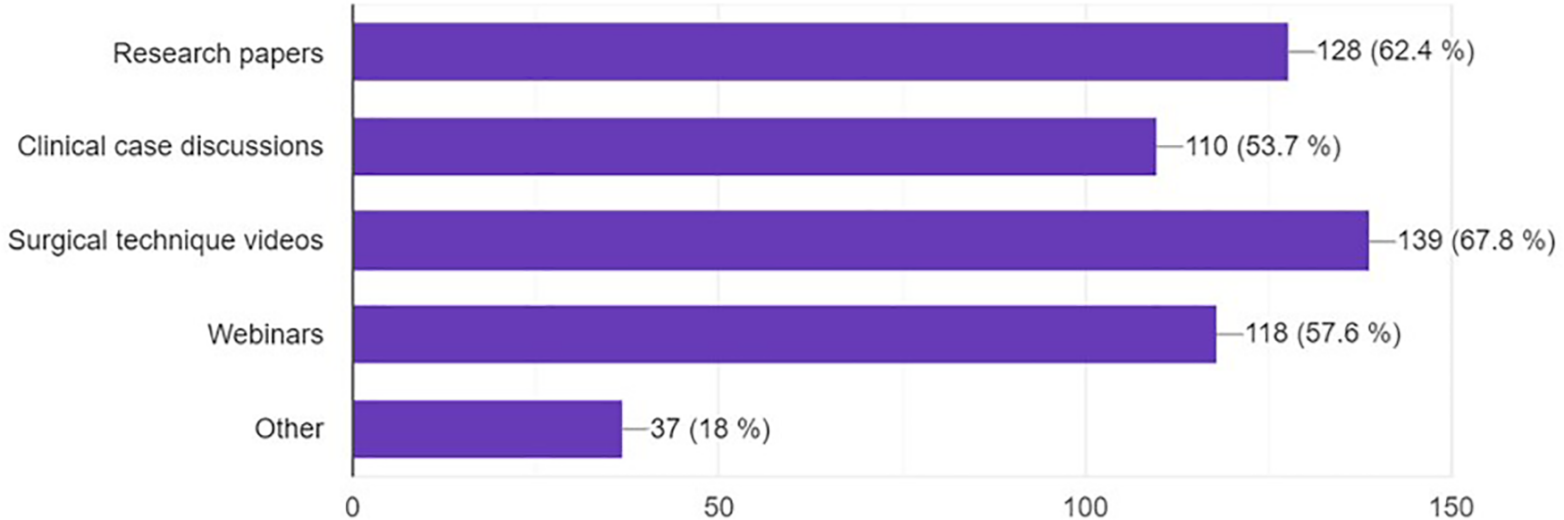
Type of content accessed on social media platforms.

The most sought-after content on social media related to neurosurgery were surgical technique videos (67.5%), research papers (62.6%), and webinars (57.6%) (Figure 6).

### Social media and professional development

70.4% of respondents rated the contribution of social media to their professional development in neurosurgery as high (scores of 4 and 5).

### Participation in online events and seeking advice

**Figure 7:**
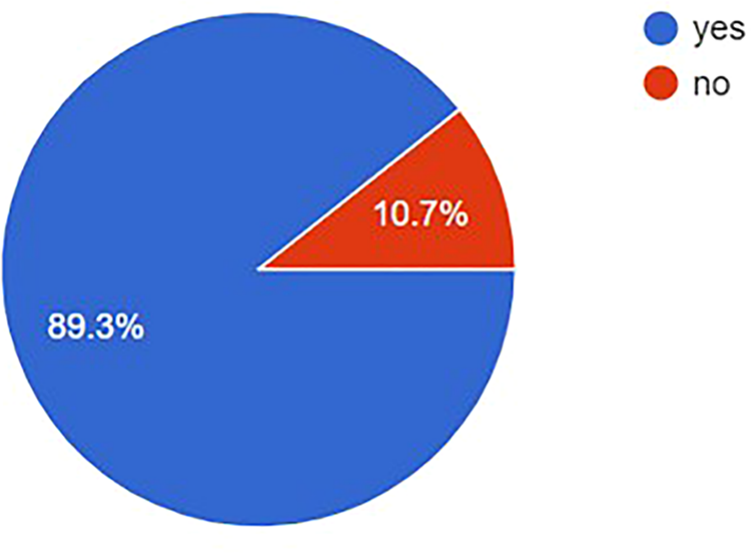
Proportional of participants accessing an online course of webinar organized via social media.

A high percentage (89.2%) reported participating in a neurosurgery webinar or online course facilitated through a social media platform (Figure 7).

Most respondents (72.2%) had sought or received advice on a challenging case from colleagues on social media.

## Concerns and ethical issues

**Figure 8:**
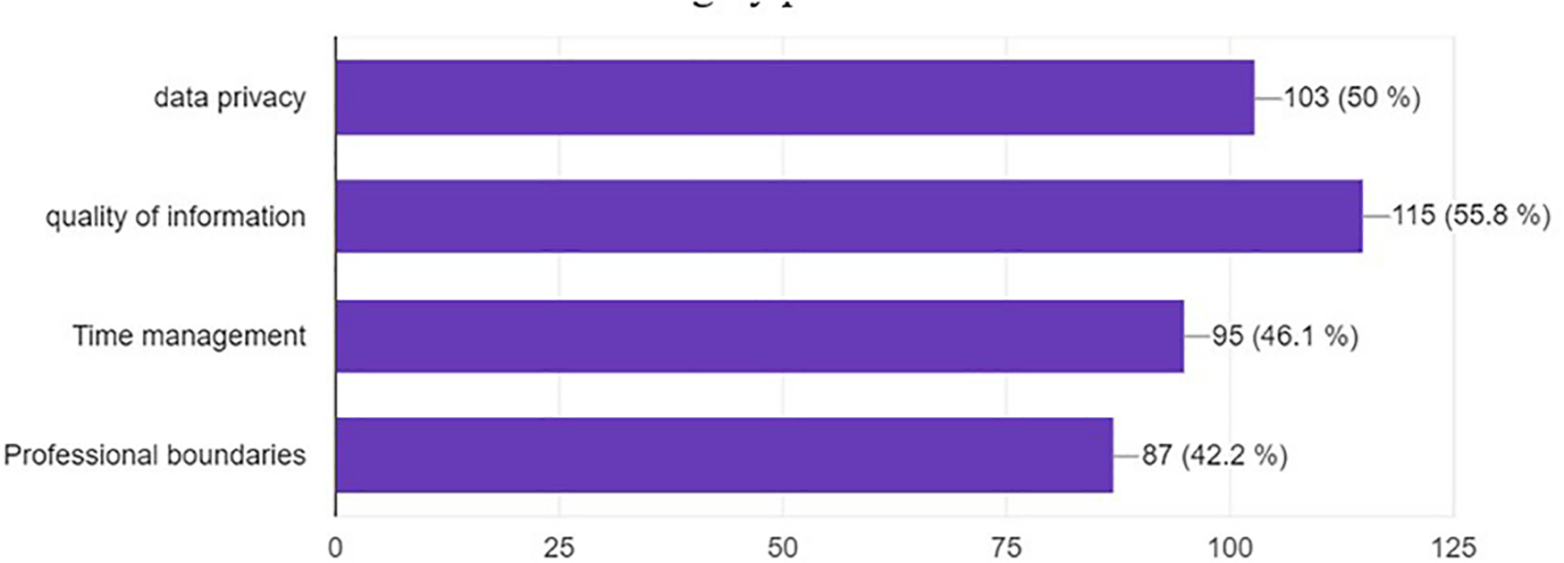
Most common concerns about social median expressed by participants.

The main concerns about the use of social media in neurosurgery training were the quality of information (55.8%), data privacy (50%), time management (45.6%), and professional boundaries (42.2%) (Figure 8).

A significant portion (72.9%) reported encountering negative consequences or ethical issues related to their use of social networks in neurosurgery practice.

### Preferred resources

**Figure 9:**
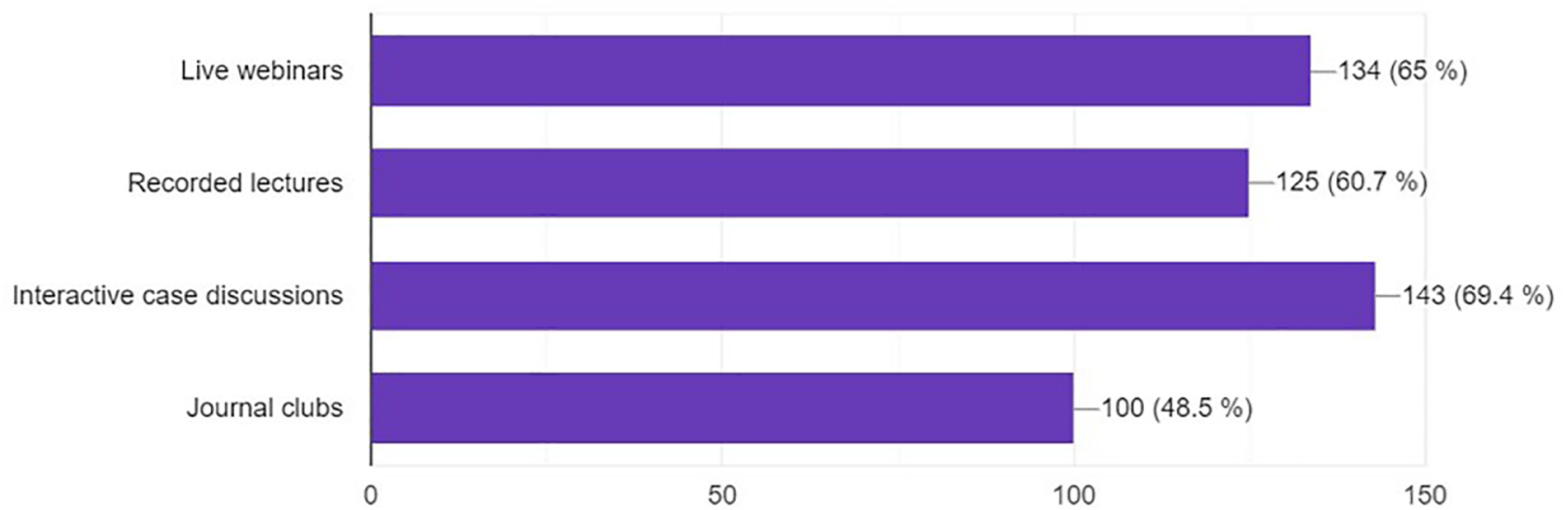
Preferred type of resources to learn about neurosurgery training.

The most preferred types of resources were interactive case discussions (69.4%), live webinars (65%), and recorded lectures (60.7%) (Figure 9).

#### Future of social media in neurosurgery training

The majority (90.8%) of respondents believed that social media has a future role in neurosurgery training.

### Benefits of social networks

**Figure 10:**
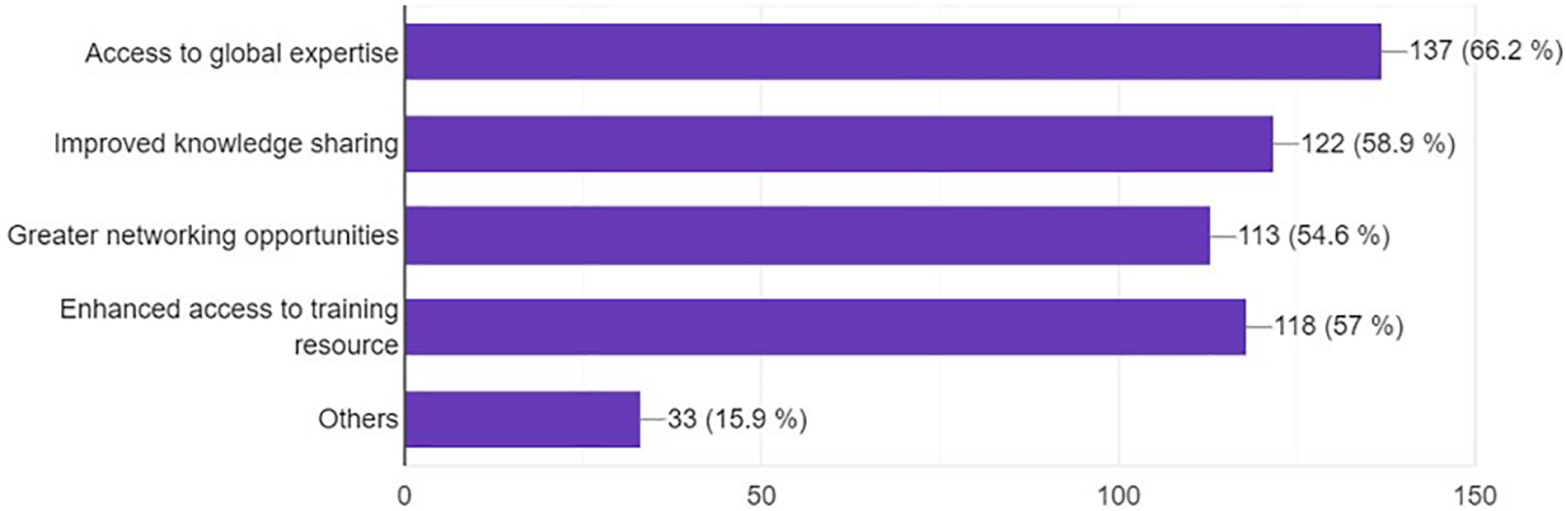
Benefits of social media expressed by participants.

The main perceived benefits of using social networks for professional purposes in neurosurgery were access to global expertise (66.2%), improved knowledge sharing (58.9%), and enhanced access to training resources (56.7%) (Figure 10).

## Discussion

Our results suggest that while social media has been beneficial for professional development and networking among neurosurgeons in LMICs, there are significant concerns around information quality, data privacy, time management, and professional boundaries. Given the lack of availability of neurosurgery training resources in many of these countries, further exploration of the best ways to leverage social media for educational purposes while addressing these concerns is warranted (4).

The given data presents insightful statistics on the role of social media in neurosurgery training. It reveals important trends concerning social media use, access to neurosurgery resources, gender distribution, experience in neurosurgery, and other relevant factors. The survey demonstrates the advantages and challenges presented by social media in the context of neurosurgical education, leading to some critical points of discussion.

The demographic data indicates a high prevalence of males (85.5%) compared to females (14.5%) in the field of neurosurgery. This suggests a gender imbalance that aligns with broader trends in the medical specialty, indicating the need for initiatives to promote diversity in this area. The diverse geographical participation, with countries like Mexico, Nigeria, and Kenya at the forefront, resonates with literature that has underscored the potential of digital platforms in bridging educational disparities. Systematic reviews have noted that in regions with resource constraints, professionals are increasingly relying on digital avenues, such as social media, to supplement their learning.

The distribution of occupations and years of experience sheds light on the representation of different career stages within neurosurgery. Interestingly, there are comparable numbers of neurosurgeons and neurosurgery residents, suggesting the potential for robust interactions between experienced practitioners and those in training. This setting offers a rich environment for peer learning and mentorship.

Regarding the setting, the majority of respondents work in a rural setting (83.6%). This is noteworthy as it indicates that even those working in areas typically associated with limited resources are finding ways to remain engaged and current in their field, possibly through social media and online resources.

An essential aspect of the survey is the perception of the availability of neurosurgery training resources. Most respondents rated their access to resources as either “poor” or “very poor”, highlighting a significant gap in the provision of critical training materials. This is an area that could be potentially addressed by the use of digital tools and platforms.

The high usage of social media for professional purposes in neurosurgery (88.6%) underscores the significant role these platforms are playing in contemporary neurosurgical education. The results also provide an understanding of the types of content most commonly sought or shared on these platforms, with “surgical technique videos”, “research papers”, and “webinars” ranking highly. This implies that social media offers an efficient medium for sharing various forms of content and encourages peer-to-peer learning.

Interestingly, despite concerns such as data privacy, quality of information, time management, and professional boundaries, most respondents (73.2%) parrticipants have reported negative consequences and ethical dilemmas stemming from the use of social networks in their practice. A primary concern is the risk of inadvertently identifying patients during neurosurgical case discussions on public or semi-public platforms. Even when names and overt details are omitted, shared images or specific case elements can inadvertently reveal a patient's identity. This risk is heightened in the diverse cultural landscapes of low/middle income countries, where the sharing of certain information might be viewed as intrusive or insensitive (16).

Further complicating the matter is the pressing need to share educational content. In the haste to disseminate knowledge, the essential step of obtaining patient consent can sometimes be sidestepped, violating not just ethical boundaries but also legal norms in various region (17).

Additionally, the security limitations of mainstream social media platforms present considerable challenges. The ease with which confidential information can be mismanaged or misconstrued is a significant worry. Absent a regulated and consistent environment, the information shared runs the risk of being misconstrued, paving the way for potential misinformation.

While the benefits of social networks for neurosurgical education in low/middle income countries are undeniable, it's imperative to approach this digital shift with caution and commitment to upholding, if not enhancing, patient confidentiality and professional integrity. The complexities associated with the use of social media in this context underscore the need for deeper investigation and proactive measures to offset potential pitfalls (18).

The future role of social media in neurosurgery training is widely accepted, with 90.7% believing it has a future. The primary benefits identified include access to global expertise, improved knowledge sharing, greater networking opportunities, and enhanced access to training resources. The challenges, especially concerns regarding information quality and data privacy, mirror the findings of Phillips et al. (2019). In their analysis, they outlined the potential pitfalls of over-reliance on social media platforms, emphasizing the need for discernment in sifting through vast amounts of information (19).

The data also throws light on the notable role of social media in neurosurgery training, particularly in the context of low and middle-income countries (LMICs). These regions often grapple with limited resources, which is reflected in the high percentage of respondents rating the availability of neurosurgery training resources in their countries as “very poor” or “poor”. Thus, the application of social media and digital platforms is even more pertinent, offering a relatively cost-effective and accessible solution to bridge this gap (12, 20).

### Limitations

This study acknowledges some limitations. Firstly, as a survey-based study, the results are subject to bias, including response bias and selection bias. Second, the survey may not have reached all practicing neurosurgeons in LMICs, Geographical disparities in the dataset, with underrepresentation from regions like Europe and Oceania, caution against universal generalizations.

Despite these limitations, this study provides valuable insights into the role of social networks in neurosurgery training in low and middle-income countries. Future research could explore the impact of specific social networking platforms on neurosurgery training and determine the best strategies for their use in enhancing neurosurgical education.

## Conclusion

Our article highlights the vital and growing role of social media in neurosurgery training and practice in LMICs. Our respondents, consisting of a majority of male practitioners from both rural and urban settings, have indicated a considerable reliance on these digital platforms for professional development, resource sharing, and collaboration. The palpable gap in access to neurosurgical training resources, particularly in certain regions, highlights the indispensable utility of social networks in bridging this divide. Surgical technique videos, research papers, and webinars emerge as the linchpins of content dissemination, attesting to the dynamic, multimedia-driven nature of contemporary learning.

Social media presents a cost-effective and far-reaching solution, providing a platform that transcends geographic and economic barriers to offer global expertise, foster greater networking opportunities, and enhance access to training resources.

Despite the advantages, it is important to consider the unique challenges that might be faced in these regions. For instance, limited internet connectivity, inadequate infrastructure to support digital platforms, or lower digital literacy rates might hamper the full exploitation of social media's potential in these areas. Furthermore, the potential for misinformation on social media platforms also poses a significant risk, especially in settings where there might be limited access to other authoritative resources for verification.

## Data Availability

The original contributions presented in the study are included in the article/Supplementary Material, further inquiries can be directed to the corresponding author.
